# An Evolutionarily Conserved Arginine Is Essential for Tre1 G Protein-Coupled Receptor Function During Germ Cell Migration in *Drosophila melanogaster*

**DOI:** 10.1371/journal.pone.0011839

**Published:** 2010-07-28

**Authors:** Angela R. Kamps, Margaret M. Pruitt, John C. Herriges, Clark R. Coffman

**Affiliations:** Department of Genetics, Development, and Cell Biology, Iowa State University, Ames, Iowa, United States of America; University of Texas MD Anderson Cancer Center, United States of America

## Abstract

**Background:**

G protein-coupled receptors (GPCRs) play central roles in mediating cellular responses to environmental signals leading to changes in cell physiology and behaviors, including cell migration. Numerous clinical pathologies including metastasis, an invasive form of cell migration, have been linked to abnormal GPCR signaling. While the structures of some GPCRs have been defined, the *in vivo* roles of conserved amino acid residues and their relationships to receptor function are not fully understood. Trapped in endoderm 1 (Tre1) is an orphan receptor of the rhodopsin class that is necessary for primordial germ cell migration in *Drosophila melanogaster* embryos. In this study, we employ molecular genetic approaches to identify residues in Tre1 that are critical to its functions in germ cell migration.

**Methodology/Principal Findings:**

First, we show that the previously reported *scattershot* mutation is an allele of *tre1*. The *scattershot* allele results in an in-frame deletion of 8 amino acids at the junction of the third transmembrane domain and the second intracellular loop of Tre1 that dramatically impairs the function of this GPCR in germ cell migration. To further refine the molecular basis for this phenotype, we assayed the effects of single amino acid substitutions in transgenic animals and determined that the arginine within the evolutionarily conserved E/N/DRY motif is critical for receptor function in mediating germ cell migration within an intact developing embryo.

**Conclusions/Significance:**

These structure-function studies of GPCR signaling in native contexts will inform future studies into the basic biology of this large and clinically important family of receptors.

## Introduction

Signaling mediated by G protein-coupled receptors (GPCRs) facilitates the transmission of extracellular environmental cues into a cell, regulating a myriad of cellular responses and signaling cascades involved in development, homeostasis, and disease states. GPCRs represent the largest class of cell surface receptors and include over 800 different receptors in humans [1], [2]. Abnormal GPCR function contributes to the onset of many pathologies including cancer, vascular, and neurodegenerative diseases. For this reason, GPCRs are one of the most common targets for pharmaceutical intervention in the treatment of human disease [3]–[5].

The rhodopsin family shares the characteristic seven transmembrane structure of GPCRs. Additionally, a highly conserved E/N/DRY (Glutamic Acid/Asparagine/Aspartic Acid-Arginine-Tyrosine) motif is found at the junction of the third transmembrane domain and second intracellular loop of these signal transduction molecules. The arginine of this triplet is a hallmark of these receptors and is conserved in 96% of rhodopsin family GPCRs [6], [7]. Substitution of this residue in tissue culture cells results in disruption of receptor signaling [8]–[10].

The well-studied chemokine receptors, including CXCR4, are among the GPCRs with the conserved E/N/DRY motif. CXCR4 receptors have established roles in tumor metastasis [11]. They have also been shown to mediate germ cell migration in zebrafish, chickens, and mammals [12]–[17]. In *Drosophila melanogaster* germ cells, the GPCR Trapped in endoderm 1 (Tre1) appears to be a functional analog to CXCR4 in vertebrates, playing a critical role in the migration of primordial germ cells [18]–[21].

The *scattershot* (*sctt*) mutation results in severe disruption of germ cell migration [22]. Here we demonstrate that *sctt* causes mis-splicing of the *tre1* transcript, resulting in an in-frame deletion of 8 codons, including two encoding the conserved arginine and tyrosine of the E/N/DRY motif. The *sctt* mutant provided a unique opportunity to perform a structure-function analysis at single amino acid resolution of this evolutionarily conserved motif within an intact developing organism. We more fully characterized the *sctt* mutant phenotype and performed a transgenic rescue analysis that demonstrates that the defects in germ cell migration observed in *sctt* mutants can be rescued by Tre1 constructs containing the arginine of the conserved E/N/DRY motif. Substitution of the arginine with an alanine without altering the other 7 amino acids results in a loss-of-function phenotype that does not rescue *sctt* mutants. This provides evidence that the arginine plays a critical role in maintaining the signaling function of this GPCR.

## Results

### The *sctt* mutation disrupts normal germ cell migration

**Figure 1:**
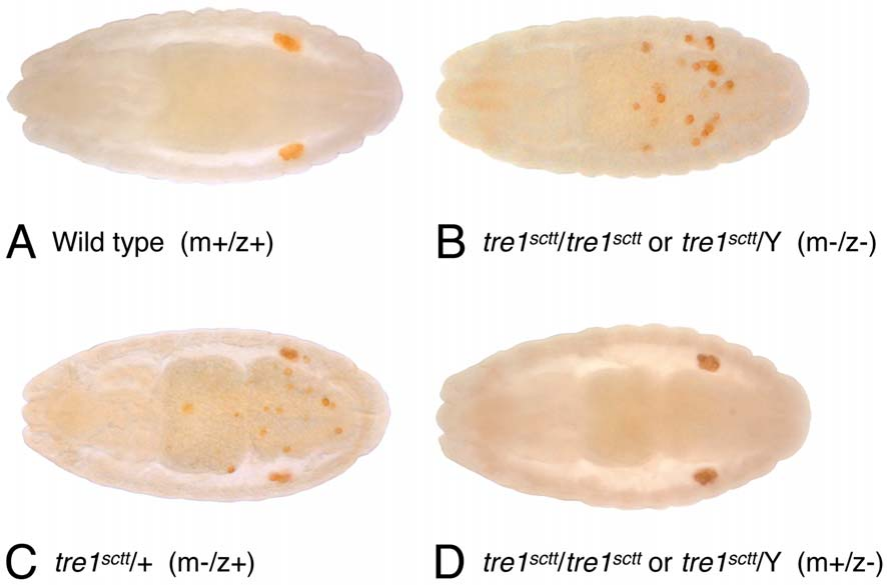
The *tre1^sctt^* mutation disrupts germ cell migration. (A-D) Dorsal views of stage 15–16 embryos are shown. Anterior is to the left. Germ cells are labeled brown with an anti-Vasa antibody. (A) In wild-type embryos, the germ cells migrate to and coalesce with the somatic gonadal precursor cells. (B) Germ cells do not migrate to the gonads in *tre1^sctt^* maternal-/zygotic- (m-/z-) embryos. (C) Germ cell migration is restored in *tre1^sctt^* maternal-/zygotic+ (m-/z+) embryos that have a wild-type *tre1* gene supplied paternally. (D) Germ cell migration is normal in *tre1^sctt^* maternal+/zygotic- (m+/z-) embryos.

In *sctt* mutants, the migration of the germ cells to the gonads is severely disrupted [22]. Germ cells fail to migrate to and coalesce with somatic gonadal precursor cells in embryos produced from a cross between a *sctt*/*sctt* female and a *sctt*/Y male (Figure 1B). Few, if any, of the germ cells reached the gonads (Table 1). The *sctt* allele is X-linked, recessive, and shows a maternal effect. One maternal copy of *sctt^+^* is sufficient to completely rescue germ cell migration (Figure. 1D). In embryos from a homozygous *sctt* mutant female, germ cell migration can be rescued with a paternally supplied wild-type copy of the *sctt* gene [22]. Embryos derived from a cross between *sctt*/*sctt* females and a *sctt* ^+^ male display two phenotypes depending upon whether they inherit the wild-type X chromosome or the Y chromosome from the paternal genome. The *sctt* maternal^-^/zygotic^+^ embryos are rescued for germ cell migration (Figure 1C), while those embryos with the *sctt* maternal^-^/zygotic^–^ background have a severe germ cell migration phenotype (Figure 1B).

**Table 1:** Germ cell distribution in *tre1^sctt^* mutants.

Genotype			Germ Cell Counts			
Maternal	Paternal	Zygotic	In Gonads	Ectopic	Total	N
wt/wt^a^	wt/Y	maternal+/zygotic+	14.7±0.4	0.5±0.1	15.2±0.4	61
*tre1^sctt^*/*tre1^sctt^*	*tre1^sctt^*/Y	maternal-/zygotic-	1.0±0.2	16.3±0.6	17.3±0.7	69
*tre1^sctt^*/*tre1^sctt^*	wt/Y	maternal-/zygotic- b	0.3±0.1	16.6±0.8	16.9±0.7	24
		maternal-/zygotic+ b	13.0±0.5	7.0±0.6	20.0±0.8	23

Germ cell counts performed on stage 15–16 embryos, Mean ± S.E.M.

a wt denotes the non-mutagenized *w^1118^, P{w^+^, fat facets-lacZ}* parental strain.

b two distinct phenotypic classes, presumed genotypes are based on genotyping experiments performed in Coffman *et al.* 2002.

To establish a baseline for future transgenic rescue experiments and to better define the scattershot phenotype, germ cell counts were performed to determine the number of germ cells that reached the gonads in various *sctt* mutant backgrounds. Germ cell counts at stages 15–16 revealed an average of 14.7 germ cells reached the gonads in wild-type embryos (Table 1). These numbers are in agreement with previously published results from other genetic backgrounds [23]–[26]. While the total number of germ cells in *sctt* maternal^-^/zygotic^–^ embryos is within the range of wild type, on average less than 1.0 germ cell per embryo reached the gonads. Rescue of germ cell migration by a paternally supplied copy of *sctt ^+^* was complete, an average of 13.0 germ cells was observed in the gonads of *sctt* maternal^-^/zygotic^+^ embryos, compared to 14.7 germ cells in the gonads for wild type. A germ cell death defect was associated with *sctt* maternal^-^/zygotic^+^ embryos. On average, there were 7.0 germ cells ectopic to the gonads in *sctt* maternal^-^/zygotic^+^ embryos compared to 0.5 germ cells ectopic to the gonads in wild-type embryos. The *sctt* maternal^-^/zygotic^-^ embryos from the same cross averaged 0.3 germ cells in the gonads (Table 1). This data supports and extends previous findings that germ cell migration to the gonads in *sctt* mutants is severely hindered but is successfully rescued when embryos express a wild-type copy of *sctt* [22].

### The *sctt* mutation is an allele of *tre1* that alters the splice acceptor site in intron 4 of *tre1*

The *sctt* maternal^-^/zygotic^-^ germ cell phenotype suggested that the molecular defect causing this phenotype represents a severe loss of function. However, the molecular lesion causing the *sctt* mutation was unknown. It was known that *sctt* was an X-linked mutation [22]. To determine the location of the *sctt* gene, the *sctt* mutant chromosome was tested for complementation by crossing it to the Bloomington series of X chromosome deletion stocks. The *sctt* chromosome complemented all available deletions. Recombination mapping of *sctt* using a *w cv wy f* mapping X chromosome placed *sctt* within 1 map unit and distal to the *crossveinless* locus at 5A13. This suggested that *sctt* might be located in the 5A4 to 5A8-9 region, the gap between Df(1)JC70 (4C11;5A4) and Df(1)C149 (5A8-9;5C5). This mapping was consistent with the observation that the translocation Dp(1;Y)dx^+^5 carrying genomic sequence from 4C11;6D8 of the X chromosome on the Y rescued the *sctt* cell migration phenotype. However, the Dp(1;Y)dx^+^1 translocation of genomic sequence from 5A8-9;6D8 failed to rescue the *sctt* defect. The proximal limit of this region, 5A8-9, as defined by polytene chromosome breakpoints was in rough agreement with the recombination mapping, although the recombination mapping suggested that the gene might be in a slightly more proximal location. As noted below, the *sctt* gene corresponded to CG3171 located at 5A11-12.

**Figure 2:**
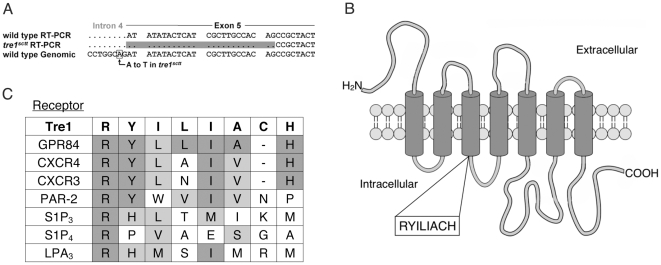
The *tre1^sctt^* mutation results in an in-frame loss of eight amino acids. (A) Reverse transcriptase PCR was performed on mRNA from 0–8 hour *tre1^sctt^* and wild-type embryos. The *tre1^sctt^* template reveals a deletion of 24 base pairs of exon 5, following the A to T base pair change in intron 4. The single base pair change is boxed. The nucleotides missing in the *tre1^sctt^* cDNA are highlighted. (B) Schematic diagram showing the predicted secondary structure of Tre1. The SOSUI, TopPred, and TMHMM all predict the eight amino acid deletion results in the shortening of the second intracellular loop. However, the overall topology of the rest of the protein is unaffected. The missing amino acids, RYILIACH, are indicated. (C) A sequence alignment comparing the *tre1^sctt^* amino acid deleted region to human GPCRs involved in cell migration. Identical residues are in dark gray and similar residues are in light gray.

Concurrent studies by Kunwar *et al.* revealed that the *sctt* chromosome failed to complement a deletion allele of *tre1*, ΔEP5 [19], [27]. However, since no molecular lesion had been identified, it remained unclear whether this represented allelic or non-allelic non-complementation. While the genetic mapping data suggested it was possible that *sctt* was an allele of *tre1,* genomic sequencing by Kunwar *et al.* found no evidence of a molecular lesion in the *tre1* coding region of the *sctt* mutant [19]. In addition, it was determined that *tre1* mRNA levels were not significantly decreased in the *sctt* background [28]. This argued that the *sctt* lesion was not resulting in a large change in *tre1* transcription. To identify if the *sctt* mutation was in *tre1*, the coding region along with introns of *tre1* was sequenced. Single adult *sctt* and wild type control flies were harvested and used for genomic templates. Primers directed at exons 2–7 of *tre1* were used in PCR to amplify and subsequently sequence this region. Nearly 2000 base pairs were sequenced on both DNA strands and a single base pair substitution was observed between *sctt* embryos and the wild type controls: an adenine within intron 4 was mutated to a thymine (Figure 2A).

The single base pair change of an adenine to a thymine within intron 4 could change the preferred splice acceptor site from AG to TG leading to improper splicing of the *tre1* RNA product. To test this, RNA was isolated from 0–8 hour old *sctt* or wild-type embryos and reverse transcriptase PCR was performed. Sequencing of the cDNA product confirmed that splicing of the wild type control occurred as predicted (Figure 2A). With the *sctt* template, intron/exon junctions through exon 4 were correctly spliced. However, directly following the altered splice acceptor site, 24 base pairs were missing from exon 5. The next suitable splice AG acceptor site in the sequence was used. This change results in an in-frame deletion of 8 amino acids, RYILIACH, from the protein (Figure 2B). The deleted amino acids are at the junction of the third transmembrane domain and the second intracellular loop of the Tre1 GPCR. Secondary structure analysis programs predict that the deletion shortens the second intracellular loop, while the remainder of the protein secondary structure is unaffected [29]–[31]. The removal of these eight amino acids from Tre1 has a significant impact on the function of the protein. This deletion includes two residues, R and Y, of the highly conserved E/N/DRY motif of rhodopsin family GPCRs. We concluded that *sctt* is an allele of *tre1*, and we will subsequently refer to it as *tre1^sctt^*.

### The arginine deleted in Tre1^sctt^ is critical for primordial germ cell migration

The severe loss of function phenotype seen in *tre1^sctt^* mutants offered a unique opportunity to perform a detailed structure-function analysis of this region of the Tre1 GPCR. Amino acid sequence comparisons of this 8 amino acid region revealed conserved residues between Tre1 and human GPCRs known to function in cell migration (Figure 2C). Because this region is conserved, it was hypothesized that some or all of the amino acids deleted in *tre1^sctt^* were critical for Tre1 function. To test the hypothesis that specific amino acids in this region were necessary for Tre1 function, transgenic constructs were created where individual or small groups of amino acids in the deleted region were replaced with alanine. These amino acid substituted constructs were engineered into the T^+^G^+^ construct that contains 10 kb of Drosophila genomic sequence including the sequence for *tre1* and an adjacent gene, *Gr5a* [27]. Transgenic lines bearing transgenes designed to express altered forms of Tre1 were established. These transgenic chromosomes were then crossed into a *tre1^sctt^*/*tre1^sctt^* genetic background. The function of the resulting Tre1 protein was assayed for maternal rescue of germ cell migration by assaying germ cell migration in embryos from a *tre1^sctt^*/*tre1^sctt^*; P[transgene] mothers that were crossed to *tre1^sctt^*/Y males.

**Figure 3:**
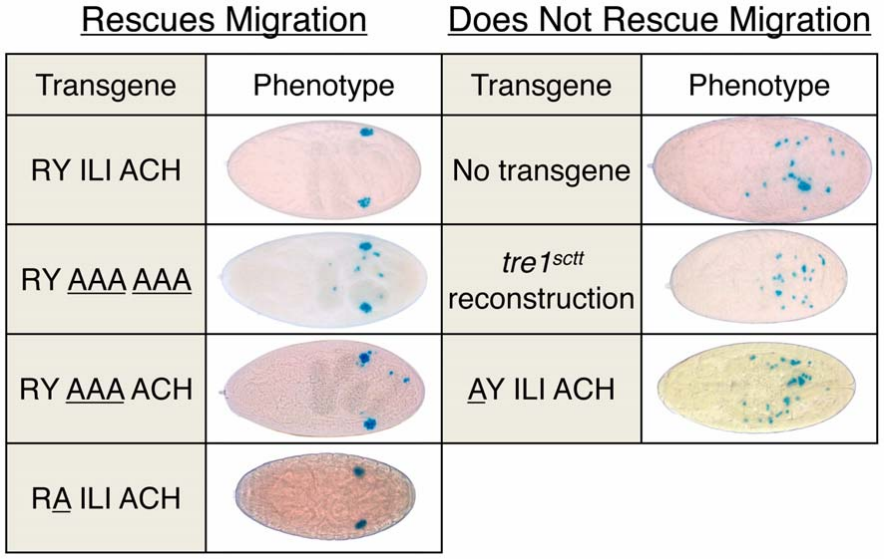
The arginine of the E/N/DRY motif is critical for Tre1 function in germ cell migration. Dorsal views of embryos are shown. Anterior is to the left. Stage 15–16 embryos were stained with X-Gal to visualize the *fat facets-lacZ* transgene, a germ cell marker. Embryos were from *tre1^sctt^* homozygous mothers containing at least one copy of the specified transgene. The substituted amino acids are underlined. Replacement of the arginine with alanine results in a transgene that fails to rescue germ cell migration in *tre1^sctt^* maternal-/zygotic- embryos. The *tre1^sctt^* reconstruction lacks the 24 base pairs missing in *tre1^sctt^* mutants.

**Figure 4:**
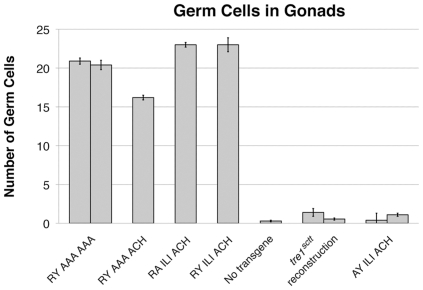
Germ cell counts showing transgenic rescue of germ cell migration in a *tre1^sctt^* mutant background. The number of germ cells in the gonads of embryos in transgenic maternal rescue of the *tre1^sctt^* defect was analyzed. All test constructs assayed rescue germ cell migration with the exception of the arginine to alanine substitution, AYILIACH. The RYILIACH construct is the positive control and the no transgene and the *tre1^sctt^* reconstruction constructs are negative controls. Error bars represent the standard error of the mean (SEM).

To verify that maternally expressed transgenes can effectively rescue germ cell migration, the T^+^G^+^ construct was tested as a positive control [27]. In embryos from *tre1^sctt^*/*tre1^sctt^;* T^+^G^+^ mothers, germ cells successfully reached the gonads (Figure 3). An average of 23.0 germ cells were observed in the gonads, compared to an average of 0.3 germ cells detected in the gonads of embryos from *tre1^sctt^*/*tre1^sctt^* females lacking any transgene (Figure 4, Table 2). These values are statistically different (P<0.0001, Student's t-test). As a negative control, a transgene that reconstructed the *tre1^sctt^* amino acid deletion was tested. A construct lacking all 8 amino acids was unable to rescue the *tre1^sctt^* defect (Figure 3). A wild-type number of germ cells were counted within the embryo, however, an average of only 1.4 germ cells reached the gonads, similar to the no transgene control (P>0.05, Student's t-test) (Table 2).

**Table 2:** Germ cell distribution in *tre1^sctt^* maternal^-^/zygotic^-^ embryos from mothers with modified *tre1* transgenes.

	Germ Cell Counts			
Transgene	In Gonads	Ectopic	Total	N
RY ILI ACH^a^	23.0±0.9	1.2±0.4	24.2±0.7	69
RY AAA AAA^b^	20.9±0.4	8.5±0.4	29.4±0.5	194
	20.4±0.6	8.9±0.7	29.3±0.9	64
RY AAA ACH	16.2±0.3	7.3±0.8	23.5±0.3	98
RA ILI ACH	23.0±0.3	1.7±0.3	24.7±0.5	64
No transgene	0.3±0.1	25.2±0.9	25.5±0.9	75
*tre1^sctt^* reconstruction^b^	1.4±0.5	23.3±0.7	24.6±0.7	103
	0.3±0.2	22.1±1.4	22.4±1.4	29
AY ILI ACH^b^	0.4±0.9	26.6±0.8	27.1±0.8	87
	1.1±0.2	17.8±0.9	18.9±0.9	77

Embryos were collected and aged to stages 15–16, Mean ± S.E.M.

Germ cells were detected by staining for ß-galactosidase activity using the *P{w^+^, fat facets-lacZ}* germ cell-specific marker.

a Wild type T^+^G^+^ vector as described in Dahanukar *et al.* 2001.

b Two independent transgenic insertions were assayed.

To identify the critical amino acids within the RYILIACH deletion, constructs were designed with combinations of the original amino acids and alanine substitutions. If the Tre1 protein is dependent on any of these specific amino acids for proper germ cell migration, their replacement with alanine should disrupt Tre1 function and these constructs should fail to rescue the *tre1^sctt^* phenotype. Alternatively, substitution of non-critical amino acids should not disrupt Tre1 function and the *tre1^sctt^* germ cell migration phenotype should be rescued.

Germ cells successfully migrated to the gonads in embryos from *tre1^sctt^*/*tre1^sctt^* mothers carrying the RYAAAAAA, RYAAAACH, and RAILIACH constructs (Figures 3 and 4, Table 2). The average number of germ cells in the gonads was 20.9 and 20.4 for the two RYAAAAAA constructs, 16.2 for the RYAAAACH construct, and 23.0 for the RAILIACH construct (Table 2). This suggests that the seven amino acids, YILIACH, are not critical for Tre1 function in germ cell migration.

When the arginine was replaced with an alanine (AYILIACH), Tre1 function was not restored (Figure 3). An average of 0.4 and 1.1 germ cells were observed in the gonads of two different transgenic lines (Figure 4, Table 2). The phenotype appears similar to the *tre1^sctt^* mutants lacking any transgene and the *tre1^sctt^* reconstruction that fails to rescue the defect (Figure 3). This demonstrates that the arginine is essential for Tre1 function. While a shortening of the second intracellular loop may also contribute to the loss-of-function observed with the *tre1^sctt^* allele, the observation that restoring the length of the loop but replacing the arginine with an alanine results in a scattershot phenotype demonstrates a clear role for the arginine of the highly conserved E/N/DRY motif in proper germ cell migration in Drosophila.

## Discussion

We report a genetic and functional analysis of Tre1, a GPCR that is essential for the migration of primordial germ cells in *Drosophila melanogaster*. Previous studies showed that the *sctt* chromosome failed to complement a chromosome carrying a deletion of *tre1* [19]. However, no associated molecular lesion had been discovered. Genomic and RT-PCR sequencing results presented in this study provide direct evidence that *sctt* is an allele of *tre1.* The *tre1^sctt^* mutation results in an AG to TG mutation at a splice acceptor site that abolishes correct splicing resulting in the utilization of a cryptic splice acceptor site and the loss of the first 24 base pairs of exon 5 (Figure 2A). Eight amino acids are missing from the third transmembrane/second intracellular loop junction of the Tre1^sctt^ GPCR, while the rest of the protein proceeds in frame. Secondary structure modeling programs predict a shortening of the second intracellular loop with the remainder of the secondary structure unaffected.

The deleted region, RYILIACH, was systematically tested for restoration of Tre1 function using an alanine scan approach. Through the design of a modular cassette vector containing the *tre1* genomic sequence, the region encoding these 8 amino acids was manipulated to insert amino acid substitutions of each amino acid. Transgenic flies created using these constructs were assayed for their ability to maternally rescue the *tre1^sctt^* germ cell migration defect in the context of developing embryos. Through this replacement approach, it was discovered that the 6 amino acids following the RY were dispensable for primordial germ cell migration. The RYAAAAAA and RYAAAACH constructs restored germ cell migration back to wild-type levels. However, maternal expression of the RYAAAAAA or RYAAAACH was not sufficient to rescue a germ cell death defect, as seven to nine germ cells were observed ectopic to the gonads (Table 2).

The E/N/DRY motif is a hallmark of rhodopsin family GPCRs that has been studied extensively in tissue culture systems [1], [9]. As shown in Figure 3, an alanine replacement of the tyrosine in this domain fully rescued the *tre1^sctt^* germ cell migration defect. It is possible that this highly conserved residue has important roles in other Tre1 functions, but it appears to be dispensable in early germ cell development. However, the neighboring arginine in Tre1 has critical roles in primordial germ cell migration. Two separate lines carrying constructs that replaced the arginine with alanine were unable to rescue the *tre1^sctt^* defect. Studies of GPCRs in other systems inform our interpretation of these results. Scheer *et al.* and Zhu *et al.* have examined the effects of analogous mutations in mammalian alpha1b-Adrenergic Receptors (R143A) and the m1 (R123N) and m2 (R121N) Muscarinic Receptors in tissue culture cells. They found that these mutations did not alter protein expression, but did impair GPCR signaling [8], [32]. We have not assayed Tre1 protein expression from these transgenes as epitope tags impaired Tre1 function (data not shown). However, other alanine substitutions within the region deleted in Tre1^sctt^ (RAILIACH, RYAAAACH, and RYAAAAAA) did provide Tre1 function. We therefore conclude that the failure of the arginine substituted construct to rescue *tre1^sctt^* loss of function is due to a lack of protein function rather than reduced expression.

The arginine of the E/N/DRY motif located towards the cytoplasmic side of the third transmembrane domain is conserved in 96% of rhodopsin family GPCRs [6], [7]. A sequence alignment of seven human rhodopsin family GPCRs involved in cell migration illustrates the invariant nature of this residue (Figure 2C). Rhodopsin family receptors are a set of highly diverse GPCRs both in their ligand binding ability and their elicited cellular responses. Cell culture experiments using nonconservative mutations of this residue result in defective signal transduction [8], [9], [32], [33]. In addition, others have suggested that this arginine can directly bind G proteins [34]. Alternatively, this arginine is proposed to be involved in the stability of receptor conformation [35]–[41]. While much research into the effects of amino acid alterations of the E/N/DRY motif has been performed in cell culture systems, this conserved motif has not been studied in the more complex and native signaling environment of an intact organism. We demonstrate that changing the arginine of this motif severely impacts the ability of this GPCR to function in primordial germ cell migration during Drosophila embryogenesis.

GPCR function has been identified as having critical roles in the directed migration of a variety of cell types. An emerging theme in cell migration is that ligands such as chemokines and phospholipids function as attractants for cells to specific locations. These ligands activate GPCRs on the receiving cell's surface to initiate a migratory response toward higher levels of the agonist. The phosopholipid sphingosine-1-phosphate receptors S1P_1–4_ have been implicated in lymphocyte recirculation and tissue homing critical in adaptive immune responses [42]. Additionally, the SDF-1/CXCR4 ligand-GPCR pair has emerged as a conserved mechanism regulating a variety of cell migrations in cancer, immune response, and in development. In breast cancer, it has been found that secondary site tumor colonization during cancer metastasis is not random, but rather there is directed migration of CXCR4-expressing cancer cells towards the SDF-1 ligand at common secondary sites such as lungs and bone marrow [43]. This common ligand-receptor pair has also been found to play a role in immune response and leukocyte trafficking to tumors [11]. Similar to Tre1, the CXCR4 receptor has roles in mouse, chick, and zebrafish germ cell migration [12]–[17], [44], [45].

Tre1 is related to GPCRs with roles in the migration of cells (Figure 2C). While previous studies have used tissue culture systems to assess GPCR structure-function relationships, detailed molecular analyses of mutated receptors have not been performed in complex and dynamic systems where the cells under study are in contact with neighboring cells and responding to endogenous extracellular signals. This study provides conclusive evidence that the arginine of the highly conserved E/N/DRY motif is critical for Tre1 GPCR-mediated germ cell migration within the context of a developing organism. Given the conserved nature of this arginine residue in other GPCRs and its identification as a critical residue from cell culture studies [8], [9], [32], [33], it is likely that this arginine is critical to the function of many other GPCRs in a wide variety of cell types and organisms.

## Materials and Methods

### Fly stocks

The *sctt* allele was generated in an EMS mutagenesis screen [22]. The T^+^G^+^ transgenic line was kindly provided by John Carlson [27], [46]. *w^1118^,* P*{w^+^, fat facets-lacZ}*, the unmutagenized parental strain of *sctt*, was used as a wild-type control [47]. Balancer containing stocks were obtained from the Bloomington Drosophila Stock Center.

### Embryo collections

Embryos were collected on standard apple juice agar plates and aged to stages 15–16 at 25 °C [48]. Embryos were harvested and dechorionated using 50% bleach.

### Whole mount antibody staining

Immunostaining was performed according to standard procedures [49]. The primary antibody used was chicken anti-Vasa, (1∶10,000, a gift from Ken Howard). The secondary antibody was biotinylated anti-chicken IgG (1∶500) (Vector Laboratories). Antibody detection was performed using Vector Laboratories ABC Elite Kits with 3,3′-diaminobenzidine tetrahydrochloride as a substrate.

### ß-Galactosidase staining

ß-Galactosidase staining of embryos followed published procedures [50], [51]. Staining was performed with a 0.08% X-Gal solution for 2 hours at 37°C.

### Germ cell counts

Germ cells were labeled using either a ß-Galactosidase assay or anti-Vasa antibody and counted using a differential interference contrast microscope. Both methods detect similar numbers of primordial germ cells [52]. Embryos were aged to stages 15–16. Staging was confirmed using embryo morphology [48]. Criteria for scoring a germ cell as in the gonad included migration to abdominal segment 5 and presence within the correct bilateral region to be incorporated into the gonad. Gonadal sheath cells were used to delimit the gonad boundaries when possible.

### Genomic sequence analysis

Genomic DNA was extracted individually from seven *sctt* and five wild-type flies using a buffer containing 10 mM Tris-HCl, 1 mM EDTA, 25 mM NaCl, and 10 mg/ml Proteinase K [53]. The entire coding region of *tre1* was PCR amplified using TripleMaster Taq DNA Polymerase (Eppendorf) in combination with the following primers: 5′- TCAAATAACCAAGCGGATGC-3′, 5′-CAAAAACGTTGAGTTAGCGCC-3′, 5′- CACATCGTTTGCTTGTTTCC-3′, 5′- GCGCAAAGATCTTGTAGTAGGC-3′, 5′- CCTGGTGATCATCGTTTCG-3′, and 5′-GACAATGCGGACTAGACTTG-3′. PCR products were sequenced on both strands using an Applied Biosystems 3730xl DNA Analyzer (Iowa State University DNA Sequencing Facility).

### Reverse transcriptase PCR

0–8 hour embryos were collected, dechorionated using 50% bleach, homogenized in Trizol (Invitrogen), and total RNA isolated. DNA was removed from the total RNA using Turbo DNase (Ambion). First strand cDNA synthesis was performed using RETROscript First Strand cDNA Synthesis Kit for RT-PCR (Ambion). PCR was performed on the cDNA template using Taq DNA polymerase (Eppendorf) with the following primers: 5′-TGCTCTTCTGCTCCTTCAGC-3′ and 5′-CCAGTGTCATTAACCCGATCA-3′. Lack of genomic contamination in the PCR amplification was confirmed using primers that spanned multiple exons, and the PCR products were sequenced.

### Protein secondary structure predictions

The Tre1 amino acid sequence of both wild type and *sctt* mutants were compared using the secondary structure prediction programs: SOSUI [30], TopPred [29], and TMHMM [31].

### Engineering of the amino acid substitution cassettes

The T^+^G^+^ vector containing a 10 kb genomic fragment coding for both *tre1* and *Gr5a* was used in the generation of the amino acid-substituted constructs [27]. A 1700 base pair fragment containing the target sequence for nucleotide replacement was excised by digesting with SphI and StuI restriction endonucleases (NEB) and cloned into a modified pSP72 vector containing an inserted StuI restriction site and lacking a PstI site. The resulting pSP72 vector containing the 1700bp *tre1* insert was subsequently digested using PstI and Bpu10I (NEB) to excise a 160 base pair fragment of *tre1* genomic DNA that housed the target sequence. Phosphorylated oligonucleotides were designed to reconstitute the 160 base pair fragment and insert an AloI restriction site directly into the target sequence region. Due to the positions of the codons in relation to the intron-exon border, two independent cassette vectors were designed with the AloI site engineered in different locations to allow the substitution of nucleotides encoding all 8 amino acids of interest. Vector sequencing confirmed the presence of the AloI restriction endonuclease sites in the reconstituted 1700 base pair subclone.

A second control cassette designed to allow the monitoring of Tre1 protein expression from the *in vivo* constructs was also created. This AloI substituted 1700 base pair clone contained three copies of a myc epitope tag [54]. The epitope tag was placed in the C-terminal tail region of Tre1, HAVQ(3x Myc)KNSINQMC. Unfortunately, even a wild-type Tre1 construct with the tag failed to rescue germ cell migration.

### Creation of constructs with amino acid substitutions in Tre1

The pSP72 vector cassettes containing the target region with the engineered AloI restriction sites were digested with AloI (Fermentas). The AloI digest removed the AloI restriction site as well as 7 base pairs 5′ and 12–13 base pairs 3′ of the restriction enzyme recognition site. A pair of complementary phosphorylated oligonucleotides was designed to contain nucleotide changes to alter the amino acid sequence in the target region (Table 3). The oligonucleotides were hybridized and ligated into the AloI-digested cassette. Sequencing confirmed the presence of the desired nucleotide substitutions. The pSP72 vector was then digested with SphI and StuI to excise the 1700 base pair fragment for insertion into the digested T^+^G^+^ vector to reconstitute the 10 kb genomic clone. The splice junctions and the amino acid substituted regions were sequenced to confirm the correct reading frame and construct composition.

**Table 3:** Synthetic oligonucleotides used for transgenic constructs to evaluate Tre1 function.

Transgene	Amino Acid Change	Phosphorylated Oligos Used for Amino Acid Changes^c^
RY ILI ACH^a^	Wild type amino acids	
*tre1^sctt^* b	Lacks 8 amino acids	5′-GTAGCGGCT------------------------GCCAGGGGATTA
		5′-CCCTGGCAG------------------------CCGCTACTCGCA
RY AAA AAA	ILI ACH replaced with 6 As	5′-GTAGCGGCTGGCGGCGGCGGCGGCGGCATATCTGCCAGGGGATTA
		5′-CCCTGGCAGATATGCCGCCGCCGCCGCCGCCAGCCGCTACTCGCA
RY AAA ACH	ILI replaced with 3 As	5′-GTAGCGGCTGTGGCAAGCGGCGGCGGCATATCTGCCAGGGGATTA
		5′-CCCTGGCAGATATGCCGCCGCCGCTTGCCACAGCCGCTACTCGCA
RA ILI ACH	Y replaced with an A	5′-GTAGCGGCTGTGGCAAGCGATGAGTATGGCTCTGCCAGGGGATTA
		5′-CCCTGGCAGAGCCATACTCATCGCTTGCCACAGCCGCTACTCGCA
AY ILI ACH	R replaced with an A	5′-GGCCTACGCGTTCAGGGTGATGCCCACCATGC
		5′-GTGGGCATCACCCTGAACGCGTAGGCCCAAGT

a Wild type T^+^G^+^ vector as described in Dahanukar *et al.* 2001.

b *tre1^sctt^* reconstruction that lacks the eight amino acids missing in Tre1^sctt^, RYILIACH.

c Underlined sequences designate the nucleotide replacements used to create the amino acid substitutions or deletions.

### Generation of transgenic flies and fly crosses

The engineered T^+^G^+^ constructs were injected into a *w^1118^* host strain using a modification of the standard transformation protocol [55], as outlined by Nicholas Gompel. P{π25.7 Δ2–3 wc} was used a transposase source [56]. Transgenic stocks were established and the insertion sites of the transgenes within the genome were determined by inverse PCR [57]. The transgenic chromosomes with the amino acid substitutions were then crossed into the *tre1^sctt^* mutant background and stable homozygous or balanced heterozygous stocks established. To test for maternal rescue of germ cell migration, females homozygous for *tre1^sctt^* and carrying one or two copies of the transgenes were crossed to *tre1^sctt^* males and the offspring were assayed for germ cell migration defects.
